# Effects of Visual Priming and Event Orientation on Word Order Choice in Russian Sentence Production

**DOI:** 10.3389/fpsyg.2019.01661

**Published:** 2019-08-20

**Authors:** Mikhail Pokhoday, Yury Shtyrov, Andriy Myachykov

**Affiliations:** ^1^Centre for Cognition and Decision Making, Institute of Cognitive Neuroscience, National Research University Higher School of Economics, Moscow, Russia; ^2^Department of Clinical Medicine, Center of Functionally Integrative Neuroscience, Aarhus University, Aarhus, Denmark; ^3^Department of Psychology, Northumbria University, Newcastle upon Tyne, United Kingdom

**Keywords:** attention, constituent ordering, Russian language, perceptual priming, event orientation

## Abstract

Existing research shows that distribution of the speaker’s attention among event’s protagonists affects syntactic choice during sentence production. One of the debated issues concerns the extent of the attentional contribution to syntactic choice in languages that put stronger emphasis on word order arrangement rather than the choice of the overall syntactic frame. To address this, the current study used a sentence production task, in which Russian native speakers were asked to verbally describe visually perceived transitive events. Prior to describing the target event, a visual cue directed the participants’ attention to the location of either the agent or the patient of the subsequently presented visual event. In addition, we also manipulated event orientation (agent-left vs. agent-right) as another potential contributor to syntactic choice. The number of patient-initial sentences was the dependent variable compared between conditions. First, the obtained results replicated the effect of visual cueing on the word order in Russian language: more patient-initial sentences in patient cued condition. Second, we registered a novel effect of event orientation: Russian native speakers produced more patient-initial sentences after seeing events developing from right to left as opposed to left-to-right events. Our study provides new evidence about the role of the speaker’s attention and event orientation in syntactic choice in language with flexible word order.

## Introduction

Every day we effortlessly produce sentences talking about objects, actions, people, and events. Producing sentences about visually perceived events requires several choices to be made by the speaker. Some of these choices refer to the selection of the syntactic structure of the produced sentence. When describing a transitive event for example, a speaker of English can choose between active and passive voice frames. In addition to the choice between structural alternatives, many languages offer their speakers the choice between different word-order options (scrambling; Gell-Mann and Ruhlen, 2011). These two processes relate to the question addressed in this paper: How does the speaker decide which particular frame to choose and how to arrange the constituents in a sentence? Here, we report the results of a sentence production study that investigated how manipulations of visual attention and event orientation affect speakers’ choice of word order in Russian – a free-order language that supports scrambling via explicit case marking and explicit constituent agreement.

In a visually situated context, the sentence production process begins with image apprehension. At this stage, input from perceptual modalities (e.g., visual, auditory, and motor) provides initial information for conceptual and linguistic interpretation of the event, with attention acting as a filter modulating and ranking the input according to what is relevant, noticeable, or important. The final product of this filtration process is then coded by the production system and is reflected in a generated sentence. Existing literature provides evidence that the speaker’s attentional state is reflected in their choice of syntactic structure (see Myachykov et al., 2018b for a recent review). In one of the earliest studies (Tomlin, 1995), English-speaking participants watched a film depicting one fish (the agent) eating another fish (the patient). Attention of the speaker was manipulated by means of an explicit (i.e., consciously processed) exogenous visual cue - an arrow pointer above either the agent or the patient. The task was to continuously describe the interaction between the two fish including the eating event itself (the *target event*). Descriptions of the target events were analyzed for their syntactic structure: participants produced more active voice descriptions (e.g., *the blue fish eats the red fish*) when the cue was on the agent fish. When, however, attention was directed to the patient fish, a passive voice description (e.g., *the red fish was eaten by the blue fish*) was more likely. This and similar findings indicate that attention to one of the interacting protagonists is reflected in the sentence production strategies, which include assigning the referents to their constituent roles in the sentence (Gleitman et al., 2007; Myachykov et al., 2011, 2012a,b, 2018a; Coco and Keller, 2012, 2015; Iwabuchi et al., 2013; Montag and MacDonald, 2014; Rommers et al., 2017; Pokhoday and Myachykov, 2018; Pokhoday et al., 2018).

At the same time, it remains unclear whether the attentional contribution to structural choice is universal across languages. After all, English is a language with a largely restricted word order while other languages (Russian, Finnish, etc.) rely upon a wider degree of word-order flexibility. This question was addressed only in a couple of existing reports (Myachykov et al., 2011; Hwang and Kaiser, 2014). One study (Myachykov and Tomlin, 2008) used a methodology similar to Tomlin (1995) studying Russian native speakers. The results indicated that, unlike their English counterparts, Russian speakers did not assign the *subject* role to the cued referent; instead, they selected it as the sentential starting point generating patient-initial or agent-initial *active-voice* word orders in both cueing conditions. One explanation for this difference is a different degree of reliance on syntactic alternations and scrambling strategies in English and Russian: While syntactic alternations (e.g., active/passive) are quite common in English, Russian uses its explicit morphology, making scrambling a more productive and more frequently used mechanism (Kolomackiy, 2009).

While this finding provided initial evidence for the role of the speaker’s attentional focus in Russian sentence production, it was confounded by methodological limitations similar to the ones pointed out by Bock et al. (2004). The most critical points were (1) the repetitive use of the event of one fish eating the other in all trials without filler materials, (2) the explicitness of the cueing manipulation – the parallel presentation of the cue and the target. In real-life communication, salience, including visual salience, can be much more subtle; hence, one may need to use equally subtle attention manipulations in order to properly understand the role of attentional focus in structural choice. In English, such modifications have been implemented in studies that successfully replicated the original findings by Tomlin using improved experimental designs (e.g., Gleitman et al., 2007; Myachykov et al., 2012a, 2018a, as well as by authors of this paper in Pokhoday et al., 2018). However, the same has never been done in studies investigating the role of attention in sentence production in flexible word-order languages.

Another important contributor to the speaker’s behavior that rarely features in sentence production studies is the asymmetry of event conceptualization. Naturally, the same event can be perceived from a variety of perspectives that have little to do with the event’s salience but rather reflect speakers’ top-down biases. Some of these top-down biases have been extensively studied. For example, conceptual accessibility – or “the ease with which the mental representation of some potential referent can be activated in or retrieved from memory” (Bock and Warren, 1985, p. 50) has been shown to bias structural choices in a manner very similar to that of attention – a more accessibly referent tends to be assigned a more prominent grammatical role in a produced sentence. Individual components that were shown to increase conceptual accessibility and bias syntactic choice include referential imageability (Bock and Warren, 1985), givenness (Bock, 1977; Arnold et al., 2000), animacy (Prat-Sala and Branigan, 2000; Christianson and Ferreira, 2005; Altmann and Kemper, 2006; Branigan et al., 2008), definiteness (Grieve and Wales, 1973), and prototypicality (Kelly et al., 1986).

Yet another top-down feature that biases speakers’ conceptualization of the described event has to do with the distribution of the thematic roles among the event protagonists. More specifically, some reports suggest that the event’s agent is more likely to be conceptualized ahead of the event’s patient and be assigned a more prominent syntactic role, e.g., that of a Subject (Kemmerer, 2012; Cohn and Paczynski, 2013). This so-called “agent advantage” was supported in a recent study by Hafri et al. (2018). In their work they tested how the role of the referent character affects performance of participants in the unrelated tasks (attending to visual features unrelated to the roles). They found that if the target referent switched from agent to patient between trials, the response time increased. These authors concluded that such pattern of results reflects the automaticity and rapidness of referent role extraction during event perception. Overall, “other” and “error” accounted for less than 2% of the total responses (for full data see Supplementary Table S1).

The mental representations of the events tend to reflect the conceptualization asymmetry described above (Santiago et al., 2010; Tversky, 2011). Santiago et al. (2010), for example, investigated the direction of mental representations of perceived events. They reported results of three experiments, which indicate that participants perceived both video events and static events on a continuum from left to right. Tversky (2011) also discussed the existence of canonical (agent on the left) and non-canonical (agent on the right) event representations. These findings suggest a degree of canonicality in event perception with the establishment of a top-down effect that can be traced in sentence production strategies. In addition, a study by Dobel et al. (2007) tested whether the event orientation effect is a result of a hemispheric specialization or a cultural preference. They compared the drawings of German (left-to-right reading and writing) and Hebrew (right-to-left reading and writing) speakers. Participants heard a sentence in which the position of agent or recipient has been manipulated, then they were to draw the event. Hebrew speakers draw left-to right events positioning the agent on the left about 30% less frequently than German speakers. Dobel et al. (2007) concluded that there exists a bias consistent with a reading direction and thus supported the cultural hypothesis (see also Maass and Russo, 2003). Similarly, a study by Esaulova et al. (2018) had German and Arabic speakers describe visually presented events with the agent positioned on the left or on the right. Arabic speakers preferred to start their descriptions with the agents on the right while their German counterparts demonstrated the opposite preference. Hence, positioning of the referents in visual scenes may be shaped by the characteristics of the particular writing system used in the speakers’ language.

Here, we address both aforementioned features – an improved control of attention in comparison with previous work and control of agent-patient asymmetry in event conceptualization – at once. In general, we predict that the left-to-right processing bias, common in left-to-right readers, will lead to faster processing and a higher probability of using the referent on the left as the sentential starting point. In addition, if event orientation is a significant contributor to syntactic choice, one would predict an interaction between the cue location and event orientation (Myachykov et al., 2007). In sum, the present study aimed at testing the degree of the perceptual visual priming effect in syntactic alternations during Russian transitive sentence production. Deeper investigation of that aspect of sentence production can hint at the existence of different language production mechanisms, in this case grammatical role assignment mechanism, between English and Russian.

## Methods

This experiment was approved by the Local Ethics Committee of the National Research University Higher School of Economics, Moscow.

### Participants

To determine the sample size we used previous research as reference. 24 participants (18 females, mean age = 21, *SD* = 1.62) recruited from the students and staff population at the HSE University took part in the study. To participate in the study, participants had to be native Russian speakers, have normal (or corrected to normal) vision, and have no language or attention-related impairments (e.g., dyslexia and ADHD). Participants received course credits or monetary remuneration for their participation. All participants gave written informed consent before taking part.

### Design

We have adopted the procedure from our previous work (Myachykov et al., 2012a,b; Pokhoday et al., 2018). Two independent variables were manipulated: Cue Location (toward the *agent* or toward the *patient*) and Event Orientation (*Agent on the left* or *Agent on the right*). This resulted in a 2 × 2 factorial design with Cue Location and Event Orientation as within-subjects/within-items factors. The dependent variable was the proportion of the sentences where Patient referent was the first element of the sentence (Patient-first sentences).

### Materials

**FIGURE 1:**
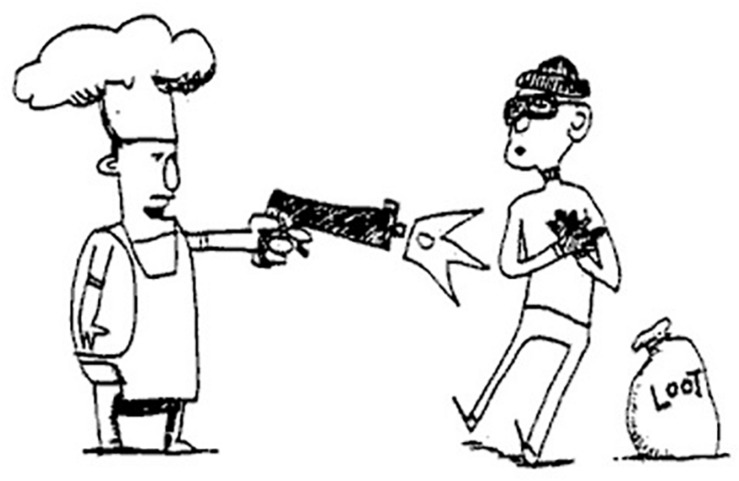
Transitive event: “The chef shoots the burglar.”

To keep experimental conditions similar to our previous studies (Pokhoday et al., 2018) we have used the same stimulus materials [adopted from Myachykov et al. (2012a, b)]. Target pictures depicted six transitive events rotated between sixteen referents (see Appendix 1 for the list of events and referents). We have crossed over the characters and the events to create 48 transitive-event target stimuli (Figure 1 for example). Each event, performed by different characters, was shown to a participant eight times. Participants received an equal number of Left-to-Right and Right-to-Left stimuli pictures. Materials were presented in a pseudo-random order such that a minimum of two filler pictures separated target pictures from each other. Filler materials (*N* = 96) were included to avoid potential structural priming bias (e.g., Bock, 1986). In filler trials, participants described ditransitive or intransitive events. In ditransitive filler trials, participants produced either double-object or prepositional-object structures. In intransitive filler trials, they produced single-referent SV sentences. Materials were arranged into four lists, which allowed all events to feature in all four experimental conditions in a fully counterbalanced fashion. Each participant saw only one list out of four.

### Apparatus

The experiment was created in SR Research Experiment Builder v2.1.140 software (SR Research Ltd., Ottawa, ON, Canada). An EyeLink 1000+ Desktop eye tracker (SR Research) was used to record fixation locations prior to presentation of a perceptual cue in order to avoid any possible directional biases. Eye movements were recorded from the right eye only with a 1000 Hz sampling rate. Stimuli were delivered by the eye tracker PC to an ASUS VG248QE 24-inch display (refresh rate 144 Hz). Generated sentences were recorded using a voice recorder application (Smart Recorder 1.8.0, SmartMob) and stored on a password protected PC. Participants were seated 60 cm away from the monitor with their head position controlled by a chinrest.

### Procedure

The study took place in the eye-tracking laboratory of the HSE Centre for Cognition and Decision Making. Before the experiment, participants provided their demographics and signed consent forms. After reading experimental instructions, participants received a practice session followed by the eye tracker calibration procedure (standard 9-point calibration, average calibration error 0.37°). The practice session consisted of two tasks. First, participants familiarized themselves with the 16 referents: the characters’ depictions were sequentially presented centrally on screen, with their names written underneath. Participants’ task was to read out loud and remember the character’s names. This ensured that participants knew the referents’ appearances and names in order to minimize cognitive effort related to recognizing the referents’ identities and retrieving their names during the main experiment. This procedure also helped to reduce potential ambiguity in naming referents [e.g., *“маляр”* (*painter*) *–* for the character *“художник”* (*artist*)]. Second, participants practiced describing events similar to the ones they would later encounter in the main experimental session. Participants saw fourteen randomly selected events in an individually randomized order, with each picture depicting an event with one or two referents (previously practiced) and the event’s name in the infinitive form [e.g., *“гнаться”* (*to chase*)] written underneath. As before, participants were instructed to examine the event and read its name aloud. The purpose of the event practice session was to minimize the variability of potential lexical candidates for the event description [e.g., *“ударить”* (*to strike*), for *“бить”* (*to hit*) event].

**FIGURE 2:**
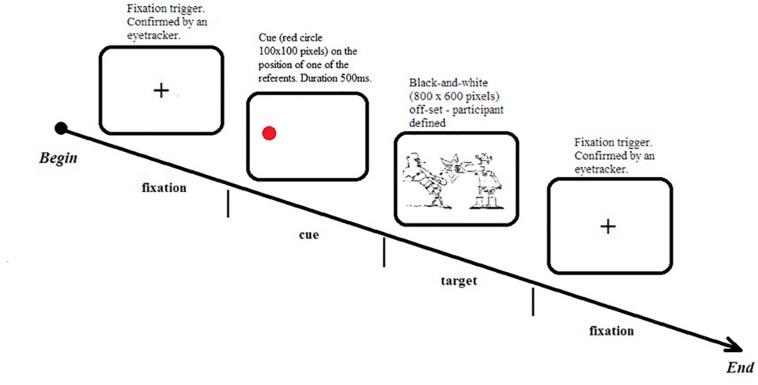
Example of the experimental trial.

Upon completion of the practice session, participants received instructions for the main part of the experiment. Participants were told that every trial would begin with the presentation of a black cross in the middle of the screen (until fixation was confirmed by the eye tracker) followed by a red circle (the cue for 500 ms) in various locations, finally followed by the presentation of a picture stimulus (until participant pressed the space bar). The cue location corresponded to the subsequent position of one of the referents. Participants were instructed to look at the black cross, then, on appearance of the red circle, direct their gaze to it, wait for the event, and then describe the event aloud in one sentence mentioning both characters and their interaction. On completion of each trial (Figure 2), participants proceeded to the next trial by pressing the spacebar.

### Data Analysis

The audio recordings of participants’ responses were transcribed and responses were coded as follows: (0) Agent First or (1) Patient First. Produced passive voice sentences (*N* = 6) were coded as Patient First sentences, as they were OVS. The responses that were not classifiable as (0) or (1) were coded as “other.” Erroneous and absent responses were coded as “error.” Overall, “other” and “error” accounted for less than 2% of the total responses.

According to the currently well-established practice we performed inferential analyses using Generalized Linear Mixed Effects Models (GLMM), as part of the lme4 package in R (R Core Team^1^). The dependent variable of interest was the use of patient initial description (True = 1 and False = 0). A binary logistic model was specified in the family argument of the glmer() function. The model included a full-factorial Cue Location (Agent, Patient) × Event Orientation (Left-to-right, Right-to-left) fixed effects design. All predictors were mean-centered using deviation-coding. We adopted the maximal random effects structure (Barr et al., 2013) justified by the design. We included in the model random correlations; by-subject and by-item random intercepts, by-subject and by-item random slopes for every main effect. These were included as both factors were within-subject and within-items. *P*-values were obtained via Likelihood Ratio Chi-Square (LRχ2) model comparisons.

## Results

Overall, 24 participants provided 1152 responses, 1131 of which were included into the analysis. The grand average intercept of the GLMM was estimated as −2.600 log odds units (SE = 0.289), which is well below zero (and in turn much smaller than 0.5 in probability space). Hence, patient-initial responses (13.5%) were greatly outnumbered by agent-initial responses (86.5%; see Table 1 for absolute counts), an expected result that is in line with previous experimental findings (Myachykov and Tomlin, 2008).

**TABLE 1:** Probabilities of agent vs. patient responses across all participants and trials (absolute cell counts in brackets) by levels of event orientation (agent-left and agent-right) and cue location (agent and patient).

**Event orientation**	**Cue location**	**Total**	**Agent initial**	**Patient initial**
Agent-left	Agent	282	0.908 (256)	0.092 (26)
	Patient	279	0.868 (242)	0.132 (37)
Agent-right	Agent	285	0.899 (256)	0.101 (29)
	Patient	285	0.779 (222)	0.221 (63)

**FIGURE 3:**
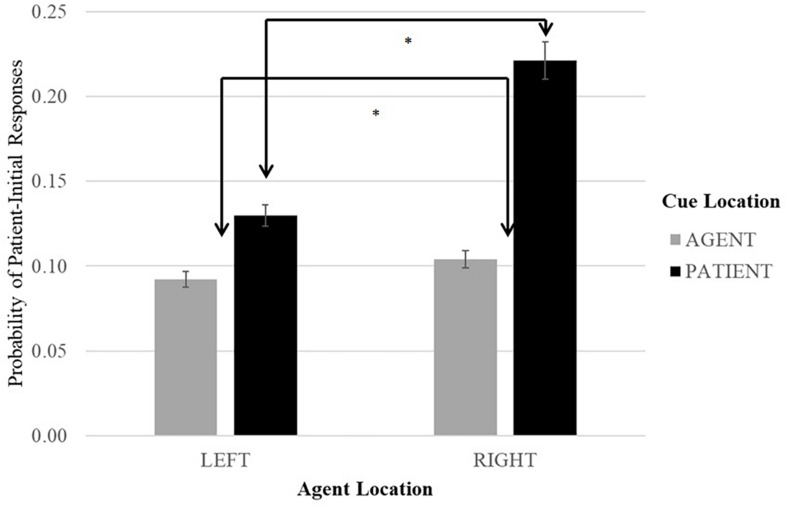
Proportion of Patient-Initial responses. Error bars represent Standard Errors. ^*^Significantly different.

Figure 3 summarizes the distribution of the patient-initial responses across experimental conditions. It is clear that, overall, there were more patient-first sentences in the patient-cued than in the agent-cued conditions. This was supported by a reliable main effect of Cue Location [_LR_χ*2*(1) = 17.268, *p* < 0.001]; the parameter estimations clarified that there were more patient-initial sentences when the patient referent was primed (*b* = −0.845, SE = 0.200, *p* < 0.001). We also registered the main effect of Event Orientation [_LR_χ*2*(1) = 5.95, *p* = 0.01]: there were more patient-initial responses when the agent was on the right side (*b* = −0.500, SE = 0.198, *p* < 0.001). Notably, there was no significant interaction between Cue Location and Event Orientation [_LR_χ*2*(1) = 2.86, *p* = 0.09; *b* = −0.694, SE = 0.398, *p* = 0.08].

In order to verify whether our sample size was adequate, we ran a *post hoc* observed power analysis. Results showed that this sample size was enough to register a moderate size priming effect (Mahowald et al., 2016). Considering the GLMM parameter estimates effect sizes of our factors of Cue location and Event Orientation were as log odds of −0.845 and −0.500, respectively. Thus, the general odds ratio effect sizes for these effects were exp(0.845) = 2.32 and exp(0.500) = 1.64. Average syntactic priming effects with and without lexical overlap reported in Mahowald et al. (2016) are 3.26 and 1.67, respectively. So, our main effect sizes are within or very close to general benchmarks of similar studies.

## Discussion

In this study, we have investigated the combined effects of perceptual priming and event orientation on the speaker’s word-order choices in Russian. Evidences suggest that perceptual priming of attention affects syntactic choice of the speaker. However, it is still unknown whether different word-order flexibility languages rely on similar mechanisms. Here, we collected data from Russian native speakers in order to assess the existence of perceptual priming effects on syntactic choice in Russian. Important addition in our study was the inclusion of event orientation in the analysis, which allowed us comparisons between bottom-up (cueing) and top-down (event orientation) priming effects. Below we discuss implications of our study.

First, we have replicated the previously reported perceptual priming effect (Myachykov and Tomlin, 2008) in a study with improved methodology and better experimental controls. We have also demonstrated that event orientation influenced syntactic choice via imposing an additional bias on the ordering of the constituents driven by the canonical left-to-right event scanning. The latter is evident as there were more patient-initial sentences when the agent was presented on the right side of the depicted event. According to some researchers, this effect might reflect the general left-to-right scanning mechanism associated with the automated writing and reading habits (e.g., Dobel et al., 2007; Santiago et al., 2010; Tversky, 2011; Esaulova et al., 2018). We did not register a reliable interaction between Cue Location and Event orientation, which suggests that the word-order choice in Russian can accommodate either the attentional (bottom-up) bias or the event orientation (top-down), but not both of these biases simultaneously. What can possibly happen is that the priming effect of the visual cue diminishes by the time structure coding occurs, while the priming effect of event orientation is present throughout all production stages due to the presence of the target stimuli picture throughout trial.

Overall, the results of the study support the hypothesis that perceptual priming influences constituent ordering but not the choice of syntactic structure in Russian. Passive-voice responses were almost non-existent in the patient-cued condition while participants still consistently encoded the cued referent as the initial element of the produced sentence. What is left unknown is whether this mechanism is similar to that of English language. As we have used Patient-initial sentences in comparison to Passive voice sentences used in English language studies, the similarity of the implied mechanism is questionable and further research is therefore necessary. Another open question is which attention network is affecting syntactic choice? This may possibly be addressed by using an Attention Network Test (Fan et al., 2005) followed in combination with stimulation of the related brain areas.

## Data Availability

All datasets generated for this study are included in the manuscript and/or the Supplementary Files.

## Ethics Statement

The National Research University Higher School of the Economics Ethics Committee Participants have signed consent forms prior to experiment. At the end of the experiment they have been debriefed.

## Author Contributions

MP contributed to write up, data collection, data analysis, and hypothesis of the manuscript. YS contributed to reviewing, editing, hypothesis, and supervision of the manuscript. AM contributed to hypothesis, reviewing, editing, analysis, and supervision of the manuscript.

## Conflict of Interest Statement

The authors declare that the research was conducted in the absence of any commercial or financial relationships that could be construed as a potential conflict of interest.

## Footnotes

1 https://www.R-project.org

## Supplementary Material

TABLE S1 Russian language data of 24 participants. DV: amount of patient-initial sentences. IV: event orientation and visual cue location.

## Appendix 1

Transitive events: hit, shoot, chase, touch, push, kick or “бить,” “стрелять,” “преследовать,” “трогать,” “толкать,” “пинать” in Russian, respectively. Referents: artist, chef, clown, cowboy, monk, nun, pirate, policeman, swimmer, dancer, professor, waitress, burglar, boxer, and soldier or “художник,” “повар,” “клоун,” “ковбой,” “монах,” “монашка,” “пират,” “полицейский,” “пловец,” “балерина,” “профессор,” “официантка,” “вор,” ”боксер,” “солдат,” in Russian, respectively.
